# Multimodal Characterization of APOE ε4‐Associated Neurovascular Heterogeneity Using Retinal, Plasma, and Neuroimaging Biomarkers in Alzheimer's Disease

**DOI:** 10.1002/cns.71220

**Published:** 2026-10-24

**Authors:** Haoran Cheng, Huwei Xia, Youjie Wang, Le Cao, Suqing Hu, Huayan Wang, William Robert Kwapong, Zhen Wang, Caiyun Wen

**Affiliations:** ^1^ Department of Geriatrics The First Affiliated Hospital of Wenzhou Medical University Wenzhou China; ^2^ Department of Neurology, Sir Run Shaw Hospital, School of Medicine Zhejiang University Hangzhou China; ^3^ Department of Neurology, West China Hospital Sichuan University Chengdu China; ^4^ Neurology Department, Xuanwu Hospital Capital Medical University Beijing China; ^5^ Department of Radiology The First Affiliated Hospital of Wenzhou Medical University Wenzhou China

**Keywords:** Alzheimer's disease, amyloid PET, APOE ε4, multimodal biomarkers, neurovascular dysfunction, PSMD, retinal imaging

## Abstract

**Background:**

Biological heterogeneity remains a major challenge in Alzheimer's disease (AD), even among individuals with biomarker‐confirmed disease. The apolipoprotein E (APOE) ε4 allele is associated with greater amyloid burden, neurodegeneration, and vascular dysfunction. We investigated whether retinal microvascular imaging provides complementary information beyond plasma and neuroimaging biomarkers for characterizing APOE ε4‐associated neurovascular heterogeneity in biologically defined AD.

**Methods:**

Eighty‐four amyloid‐positive patients with AD underwent color fundus photography, swept‐source optical coherence tomography angiography (OCTA), plasma biomarker assessment, amyloid positron emission tomography, and magnetic resonance imaging. Retinal vascular parameters were compared between APOE ε4 carriers and non‐carriers using generalized estimating equations. Candidate multimodal biomarkers were identified using least absolute shrinkage and selection operator (LASSO) regression, followed by multivariable logistic regression to evaluate the incremental contribution of retinal biomarkers within a multimodal phenotyping model.

**Results:**

Compared with non‐carriers, APOE ε4 carriers demonstrated significantly lower retinal superficial vascular complex (SVC) and deep vascular complex densities, reduced retinal vascular fractal dimension, higher amyloid PET standardized uptake value ratios, increased peak width of skeletonized mean diffusivity, and smaller hippocampal volumes (all *p* < 0.05). Following penalized feature selection, retinal SVC density remained independently associated with APOE ε4 status alongside amyloid burden, hippocampal volume, and white matter microstructural injury. Incorporation of SVC modestly improved discrimination and calibration of the multimodal model compared with neuroimaging biomarkers alone.

**Conclusions:**

Retinal microvascular alterations are associated with APOE ε4‐related biological heterogeneity in biomarker‐confirmed AD. Retinal vascular imaging provides complementary information beyond established plasma and neuroimaging biomarkers and may enhance multimodal characterization of neurovascular phenotypes rather than serve as a surrogate for genetic testing.

## Graphical abstract

The LASSO and logistic regression model integrate neuroimaging measures and retinal vascular biomarkers to identify key predictors of APOE ε4 status, improving genotype‐specific stratification. The model's calibration curve and decision curve analysis demonstrate improved discrimination and clinical utility when retinal vascular data are integrated with neuroimaging measures, offering a more accurate tool for clinical and research applications.

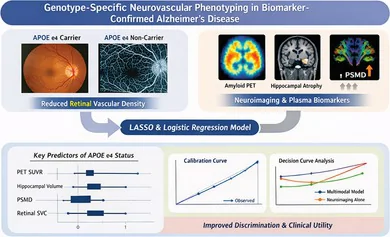

## 1 Background

Alzheimer's disease (AD) is biologically defined by amyloid‐β (Aβ) deposition, tau pathology, and neurodegeneration, as articulated in the research framework of the National Institute on Aging and the Alzheimer's Association (NIA‐AA) [1]. Within this paradigm, multimodal biomarkers—including amyloid positron emission tomography (PET), structural magnetic resonance imaging (MRI), diffusion tensor imaging (DTI), and increasingly blood‐based assays—have transformed disease characterization across the AD continuum [2, 3]. Despite sharing these core pathological features, individuals with biologically confirmed AD exhibit considerable heterogeneity in disease expression, progression, and vascular involvement [1, 4]. Understanding the biological mechanisms underlying this heterogeneity is increasingly recognized as an essential step toward precision medicine in AD.

The apolipoprotein E (APOE) ε4 allele is the strongest common genetic risk factor for sporadic AD and is associated with earlier amyloid accumulation, accelerated neurodegeneration, greater cerebrovascular dysfunction, and poorer cognitive outcomes [5, 6, 7]. Beyond its effects on protein aggregation, APOE ε4 contributes to endothelial dysfunction, blood–brain barrier impairment, and small vessel injury, implicating vascular mechanisms as integral to genotype‐specific disease expression [5, 7]. Collectively, these observations suggest that APOE ε4 influences disease expression through both neurodegenerative and vascular pathways, resulting in a distinct biological phenotype among individuals with AD.

Current multimodal biomarkers characterize complementary aspects of this phenotype. Amyloid positron emission tomography (PET) quantifies cerebral amyloid burden, hippocampal volume reflects medial temporal neurodegeneration, diffusion tensor imaging (DTI)‐derived peak width of skeletonized mean diffusivity (PSMD) provides a sensitive marker of diffuse cerebral small vessel injury, and plasma biomarkers such as Aβ42/40 and phosphorylated tau reflect central amyloid and tau pathology [1, 5, 8, 9, 10]. Although these biomarkers have substantially improved biological characterization of AD, they provide limited information regarding peripheral microvascular alterations that may accompany APOE ε4‐associated vascular dysfunction.

The retina offers a unique opportunity to evaluate systemic neurovascular integrity because of its shared embryological origin, microvascular architecture, and physiological characteristics with the brain [11]. Advances in optical coherence tomography angiography (OCTA) and quantitative color fundus photography (CFP) allow rapid, non‐invasive assessment of retinal microvascular density and vascular complexity. Growing evidence demonstrates that retinal microvascular abnormalities are associated with cerebral small vessel disease (SVD), cognitive impairment, and AD, suggesting that retinal imaging may provide complementary information regarding vascular injury occurring within the central nervous system [12, 13, 14, 15, 16, 17, 18, 19, 20, 21, 22, 23, 24].

Given the established role of APOE ε4 in vascular dysfunction, retinal microvascular alterations may represent an accessible peripheral manifestation of genotype‐associated neurovascular injury. Rather than serving as a substitute for genetic testing, retinal vascular biomarkers may provide additional biological information that complements established neuroimaging and plasma biomarkers, thereby improving characterization of the neurovascular heterogeneity present among patients with biologically confirmed AD.

Therefore, the present study investigated whether APOE ε4 carriers exhibit a distinct multimodal neurovascular phenotype characterized by convergent abnormalities in retinal microvasculature, SVD integrity, neurodegeneration, and amyloid pathology. Using APOE ε4 as a biologically defined stratification factor, we evaluated the incremental contribution of retinal vascular measures beyond established plasma and neuroimaging biomarkers in characterizing genotype‐associated biological heterogeneity. We hypothesized that retinal microvascular biomarkers would provide complementary information regarding vascular dysfunction that is not fully captured by conventional neuroimaging or blood‐based biomarkers, thereby supporting multimodal biological phenotyping of AD.

## 2 Methods

### 2.1 Study Participants

We identified participants from the prospective observational studies of AD at the Geriatrics Department of the First Affiliated Hospital of Wenzhou Medical University between September 2025 and January 2026. The clinical diagnosis of AD was based on a comprehensive clinical evaluation conducted by medical practitioners, and participants were required to meet the National Institute on Aging–Alzheimer's Association (NIA–AA) diagnostic criteria [25]. The inclusion criteria for the participants were: (1) presence of clinical dementia with a positive amyloid‐β PET scan and an MMSE score below 26; (2) availability of complete and detailed clinical data; (3) normal optic disc and retinal morphology on ophthalmic imaging and examination; and (4) ability to cooperate with and complete MRI, OCT/OCTA examinations and blood plasma analyses.

The exclusion criteria for AD patients were: (1) diagnosis of other dementia subtypes, including dementia with Lewy bodies, frontotemporal dementia, or vascular dementia; (2) current or prior ocular conditions that could influence retinal measurements, such as diabetic retinopathy, optic neuropathy, or advanced cataract; (3) history or presence of other neurological or psychiatric disorders, including stroke or schizophrenia; (4) history of vascular diseases, including poorly controlled hypertension or diabetes mellitus; and (5) history of malignancy.

Legal guardians and/or participants provided written informed consent before enrollment, and protocols were approved by the institutional review board at the First Affiliated Hospital of Wenzhou Medical University. The study followed the Strengthening the Reporting of Observational Studies in Epidemiology (STROBE) reporting guideline and Declaration of Helsinki.

### 2.2 Magnetic Resonance Imaging (MRI)

All participants underwent MRI scanning using a standardized protocol on a 3.0‐T system (SIGNA MR, GE Healthcare, WI, USA). The imaging protocol included T1‐weighted, T2‐weighted, three‐dimensional fluid‐attenuated inversion recovery (3D FLAIR), and diffusion‐weighted imaging (DWI) sequences as previously reported [5, 19, 21]. For the present analysis, high‐resolution T1‐weighted images were obtained with a magnetization‐prepared rapid gradient‐echo (MPRAGE) sequence using the following parameters: repetition time (TR) of 2000 ms, echo time (TE) of 2.19 ms, inversion time of 900 ms, field of view of 240 × 240 mm^2^, acquisition matrix of 256 × 256, flip angle of 8°, and an isotropic voxel size of approximately 0.9 × 0.94 × 0.94 mm^3^. The DTI was acquired with a spin‐echo echo‐planar imaging sequence, with 64 noncollinear gradient directions (*b* = 1000 s/mm^2^) and eight additional volumes without diffusion weighting (*b* = 0 s/mm^2^). Hippocampal volumes were quantified from T1‐weighted images through automated segmentation techniques.

#### 2.2.1 Visual Rating of Cerebral Neurodegeneration

Medial temporal lobe atrophy was evaluated using the MTA rating scale, which assigns scores from 0 to 4, where 0 indicates no detectable atrophy and 4 denotes severe atrophy [26] (Figure S1). Higher scores reflect greater structural loss, determined by reductions in hippocampal volume and enlargement of the adjacent cerebrospinal fluid spaces.

#### 2.2.2 Quantification of PSMD

The preprocessing of DTI data and estimation of the PSMD have been reported in our previous study. Briefly, denoising and Gibbs ring removing implemented in MRtrix3 were first performed [27]. An undistorted *b* = 0 image derived from the Synb0 for Diffusion Distortion Correction (Synb0‐DISCO) pipeline was used to correct for EPI distortions using FMRIB Software Library (FSL)'s TOPUP and eddy commands [28]. Subsequently, the preprocessed DTI datasets were used to calculate the PSMD value through an open‐source script (https://github.com/miac‐research/psmd). PSMD data were expressed in units of 10^−4^ mm^2^/s.

### 2.3 PET Imaging

PET scans were performed with the uMI Panorama 35S PET/CT system. ^18^F‐Flobetapir positron emission tomography (PET) scans were acquired and subjected to visual assessment for initial confirmation of Aβ deposition. Following the acquisition, the data underwent reconstruction using a 3D ordered‐subset expectation maximization algorithm. The images were visually evaluated by a nuclear medicine specialist. PET images were reconstructed with a matrix size of 192 × 192, a field of view of 350 mm, and a voxel size of 1.8 × 1.8 × 2.8 mm^3^. The ^18^F‐AV45 PET images were converted to SUVR maps with reference to the cerebellar cortex as previously reported [29].

### 2.4 Blood Plasma Analyses

Blood samples were obtained in the morning; participants were not required to fast. Venous blood was collected into six K2‐EDTA tubes and delivered to the laboratory for processing within 3 h of collection. Samples were centrifuged at 2000 × ***g*** at 4°C for 10 min. The resulting plasma was then divided into 1.5 mL polypropylene aliquots (1 mL each) and stored at −80°C until analysis. Plasma collection occurred within 1 month of the neuroimaging session. All plasma assays were conducted by Nanjing Vazyme Biotech Co. Ltd. (China) as described in our previous studies [5, 21].

On the day of analysis, plasma aliquots were allowed to thaw at room temperature. To prevent potential effects of repeated freeze–thaw cycles, only samples that had not been previously thawed were analyzed. Concentrations of Aβ42, Aβ40, NfL, p‐tau181, and p‐tau217 were quantified using 0.5 mL plasma aliquots. Analyses were carried out on the fully automated Elf S240 chemiluminescence immunoassay system (Vazyme, Nanjing, China) with the following commercial kits: Vazyme β‐Amyloid 1–40 Plasma, β‐Amyloid 1–42 Plasma, p‐tau 181 Plasma, p‐tau 217 Plasma, and NfL Plasma. All assays were conducted in accordance with the manufacturer's instructions, including post‐thaw vortexing and centrifugation steps to reduce potential interference from fibrin residues.

### 2.5 Retinal Imaging via Color Fundus Photography

Macula and optic disc‐centered photographs were taken of each eye with a digital camera (CR‐2 Digital Retinal Camera, Canon Medical Systems, USA). For all participants, macula‐centered images (2576 × 1934 pixels) from both eyes were obtained. All photographs were assessed by a trained, certified ophthalmologist masked to participant characteristics.

In this study, a methodology utilizing the MATLAB engine invoked from Python was employed to extract retinal vascular metrics as previously reported [30]. Retinal vascular fractal dimension (FD) was calculated from skeletonized line tracing using the box‐counting method, a global measure summarizing the entire branching pattern and complexities of the retinal microvasculature (Figure S1). Smaller values indicate a sparser retinal microvasculature. The reliability of the algorithm used in quantifying retinal microvasculature was described in a previous study [30, 31].

### 2.6 SS‐OCT/OCTA Acquisition and Processing

Retinal imaging was carried out using a swept‐source optical coherence tomography system (VG200; SVision Imaging, Henan, China; software version 2.0.106) as previously reported [16, 32, 33, 34, 35, 36, 37, 38]. The device employs a swept‐source laser with a central wavelength of 1050 nm and an acquisition speed of 200,000 A‐scans per second. Structural OCT images were obtained using 18 radial scan lines, each composed of 2048 A‐scans. An integrated eye‐tracking system was used to reduce motion‐related artifacts arising from blinking or head movement. For all enrolled participants, macular scans covering a 6 × 6 mm area were acquired.

Optical coherence tomography angiography (OCTA) images were acquired using a raster scanning protocol consisting of 512 horizontal B‐scans over a 6 × 6 mm^2^ region centered on the fovea. Each B‐scan included 512 A‐scans and was repeated eight times for averaging. Only angiograms with a signal quality score of 8 or higher were included in the analysis. En face angiograms of the superficial vascular complex (SVC) and deep vascular complex (DVC) were generated using automated layer segmentation to assess retinal perfusion. The boundary between the SVC and DVC was defined at the interface between the inner two‐thirds and outer one‐third of the GCIPL (Figure S1). Retinal microvascular status was quantified using vessel density (%). The reliability of the OCTA parameters applied in this study has been validated previously [39]. All angiograms used for processing followed the OSCAR‐MP Consensus Criteria for Quality Assessment of OCTA [40] and the APOSTEL recommendation [41].

For retinal imaging, images affected by ocular conditions that could compromise retinal measurements, including moderate‐to‐severe cataract, optic neuritis, or age‐related macular degeneration, were excluded. When only one eye was affected, data from the contralateral eye were analyzed; participants with bilateral pathology were excluded entirely.

### 2.7 Outcome

Apolipoprotein E ε4 levels were measured and determined. Blood samples were drawn, and the APOE ε4 genotype was determined based on the presence or absence of APOE ε4. Samples were categorized as: 1. Present (APOE ε4 with APOE ε3 or ε4); 2. Absent (no APOE ε4 or APOE ε4 with APOE ε2).

### 2.8 Statistical Analyses

Statistical analyses were conducted using R software (version 4.4.3). Statistical significance was defined as a two‐tailed *p*‐value < 0.05. Variable distributions were assessed through visual inspection and the Kolmogorov–Smirnov test. Normally distributed continuous variables are reported as mean ± standard deviation (SD), whereas non‐normally distributed variables are summarized as median with interquartile range (IQR). Categorical data are presented as counts and percentages.

Because APOE ε4 carriers and non‐carriers differed in baseline cognitive performance, additional sensitivity analyses were performed to evaluate the potential influence of cognitive status and clinical disease characteristics on the observed associations. For the comparison of retinal vascular measures, generalized estimating equation (GEE) models were additionally adjusted for hyperlipidemia, disease duration, and MoCA score. For plasma and neuroimaging measures, additional models incorporated disease duration and MoCA score as covariates. For the multivariable logistic regression models based on LASSO‐selected features, additional adjustment was performed for hyperlipidemia, disease duration, and cognitive performance (MoCA). The corresponding adjusted regression estimates are presented in Table S2. Model discrimination was additionally evaluated after adjustment for hyperlipidemia and cognitive performance, with the corresponding ROC analyses presented in Table S3. These analyses were considered sensitivity analyses to assess the robustness of the primary associations rather than as longitudinal analyses of cognitive or disease progression.

Comparisons of retinal imaging parameters between APOE ε4 carriers and non‐carriers among AD were performed using GEE models. Since measurements were obtained from both eyes of each participant and thus are correlated within individuals, analyses were conducted at the eye level with participant ID treated as the clustering factor. An exchangeable correlation structure was specified to model inter‐eye dependence, and robust standard errors were used to ensure valid statistical inference.

$$\begin{array}{c} \text{Primary}\;\mathrm{GEE}\;\text{model}:\mathrm{age}+\mathrm{sex}+\text{hypertension}\\ \;+\text{diabetes mellitus}+\text{education}+\text{intereye dependency} \end{array}$$

$$\begin{array}{c} \text{Sensitivity}\;\mathrm{GEE}\;\text{model}:\text{primary covariates}\\ \;+\text{hyperlipidemia}+\text{disease duration}+\text{MoCA}. \end{array}$$

To select potential predictive features from our study participants, we employed the least absolute shrinkage and selection operator (LASSO) regression, a robust method for handling high‐dimensional data. The optimal value of *λ* was determined using fivefold cross‐validation. Subsequently, we applied a multivariable logistic regression model to identify significant predictors, which were then used to create a nomogram. The identified features were reported as odds ratios (OR), 95% confidence intervals (95% CI), and *p*‐values.

To evaluate the nomogram's overall discriminatory ability and calibration, we plotted the calibration curve. The Hosmer–Lemeshow test was also conducted, with a significant result suggesting poor model calibration. In addition, we calculated Harrell's concordance index (C‐index) and performed bootstrapping with 1000 replicates. To assess the clinical utility of the APOE ε4 nomogram, decision curve analysis (DCA) was carried out to estimate the net benefits at various threshold probabilities within our study cohort.

Cumulative scores for each patient were calculated using the nomogram. The optimal threshold value, or cutoff, was determined by maximizing the Youden index (sensitivity + specificity − 1) through receiver operating characteristic (ROC) curve analysis. The model's accuracy was assessed using metrics such as sensitivity, specificity, predictive values, and likelihood ratios.

## 3 Results

### 3.1 Baseline Characteristics

The inclusion and exclusion criteria of our study participants are shown in Figure S2. Table 1 shows the demographics, clinical, neuroimaging, and retinal imaging measures of our study participants. A total of 84 ad was included in our final data analyses (mean age: 67.35 ± 6.56 years; 32.14% males). The median disease duration was 3 years (IQR, 2–4 years). Of note, 42 were APOE ε4 carriers while 42 were non‐carriers. APOE ε4 carriers and non‐carriers were comparable in age, sex distribution, and years of education. Vascular risk factor burden did not differ significantly between groups (Table S1).

**TABLE 1:** Demographics and clinical information of AD patients.

	AD, *N* = 84
Age, years	67.35 ± 6.56
Males, *n* (%)	27 (32.14%)
Duration, years	3 [2, 4]
Hyperlipidemia, *n* (%)	34 (40.48%)
Diabetes mellitus, *n* (%)	23 (27.38%)
Hypertension, *n* (%)	33 (39.29%)
Education, years	9 [6, 10]
*Global cognitive measures*	
MMSE	19 [14, 21]
MoCA	12 [9, 15]
*Blood plasma biomarkers*	
Aß_42_, pg/mL	6.51 ± 1.80
Aß_42/40_	0.05 ± 0.02
p‐tau 181, pg/mL	9.51 ± 4.09
p‐tau 217, pg/mL	10.23 ± 3.95
p‐tau 217/Aß_42_	1.81 ± 1.14
NfL, pg/mL	69.78 ± 49.97
GFAP, pg/mL	258.59 ± 124.47
*Retinal imaging metrics*	
Fundus vessels, FD	1.30 ± 0.11
SVC, %	37.39 ± 5.13
DVC, %	48.11 ± 6.99
*Quantitative PET measure*	
SUVR	1.23 ± 0.17
*MRI measures*	
MTA	2 [1, 2]
Hippocampal volume, mm^3^	5.78 ± 0.83
PSMD, ×10^−4^ mm/s	2.85 ± 0.48

Abbreviations: DVC, deep vascular complex; MMSE, Mini‐Mental State Examination; MoCA, Montreal Cognitive Assessment; NfL, neurofilament light chain; PSMD, peak width of skeletonized mean diffusivity; SUVR, standardized uptake value ratio; SVC, superficial vascular complex.

### 3.2 Central Neuroimaging Biomarkers

APOE ε4 carriers demonstrated significantly higher global cortical amyloid PET SUVR compared with non‐carriers (*p* < 0.001). Medial temporal neurodegeneration was more pronounced in carriers, reflected by reduced hippocampal volume after intracranial volume adjustment (*p* < 0.001). White matter microstructural injury, quantified by PSMD, was significantly elevated in ε4 carriers (*p* < 0.001, Table S1), indicating greater diffuse small vessel‐related white matter damage. After additionally adjusting for hyperlipidemia and cognitive performance (MoCA), a significant difference remained (all *p* < 0.001).

### 3.3 Plasma Biomarkers

Plasma biomarkers of amyloid and tau pathology were directionally consistent with central imaging findings. APOE ε4 carriers exhibited lower plasma Aβ42 and higher phosphorylated tau concentrations compared with non‐carriers (*p* < 0.05). Plasma neurofilament light levels showed no significant between‐group difference after covariate adjustment, as shown in Table S1. After additionally adjusting for duration and cognitive performance (MoCA), significant difference remained (Aβ42, *p* < 0.001; p‐tau 217, *p* = 0.001).

### 3.4 Retinal Vascular Measures

Figure 1 shows the comparison of retinal imaging measures between APOE ε4 carriers and non‐carriers. We showed that retinal microvasculature assayed on CFP was significantly sparser (1.35 ± 0.08 vs. 1.26 ± 0.11, *p* < 0.001), while SVC (40.84% ± 3.21% vs. 33.83% ± 4.24%, *p* < 0.001) and DVC (51.06% ± 3.55% vs. 45.06% ± 8.30%, *p* < 0.001) densities were significantly lower in carriers compared to non‐carriers. After additionally adjusting for hyperlipidemia, duration, and cognitive performance (MoCA), significant differences remained in the SVC (*p* < 0.001), DVC (*p* < 0.001), and retinal microvasculature assayed on CFP (*p* = 0.003).

**FIGURE 1:**
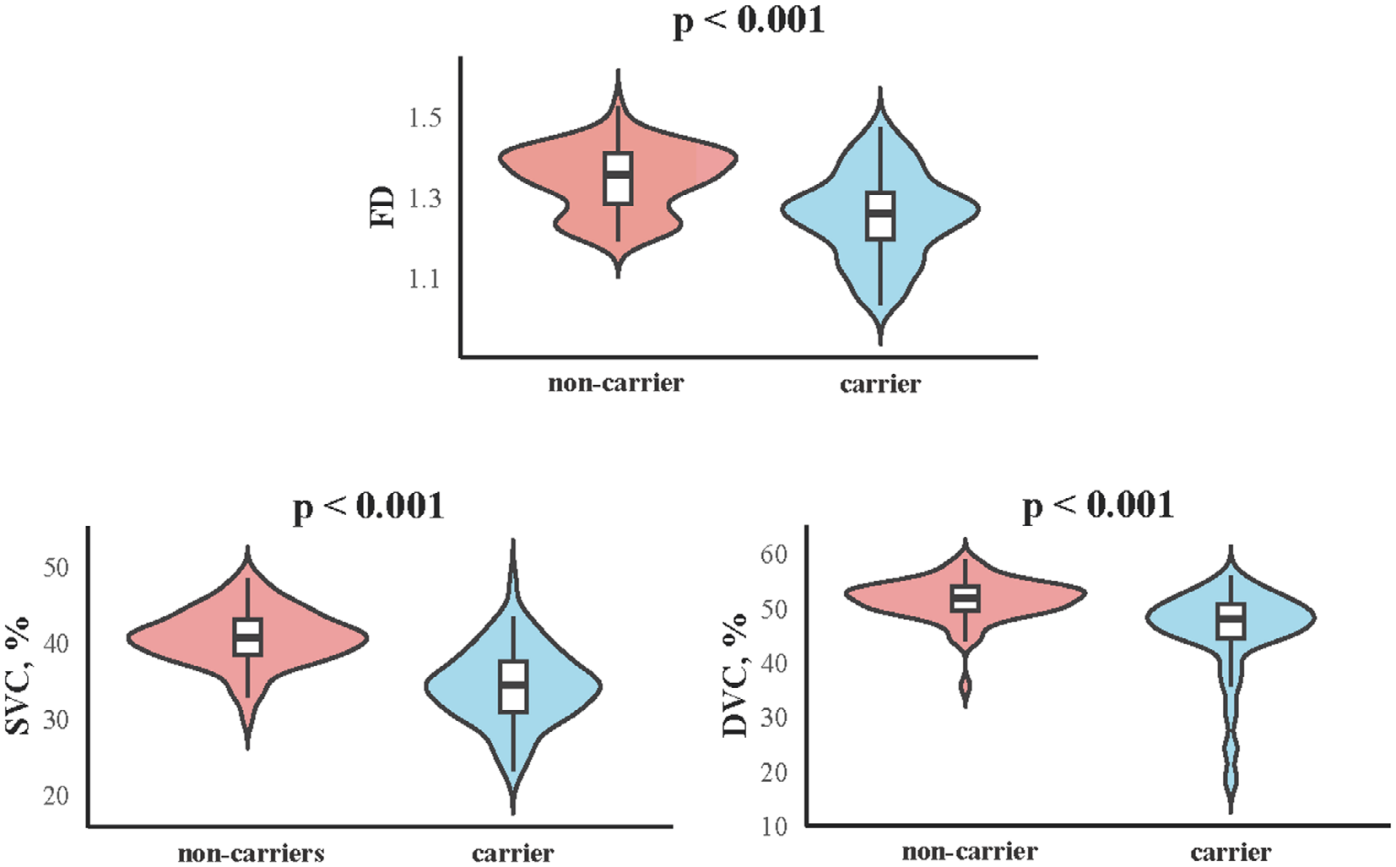
Comparison of retinal vasculature in AD with APOE ε4 carriers and non‐carriers. APOE ε4 carriers showed significantly sparser retinal vasculature (fractal dimension, FD) and reduced densities in the superficial vascular complex (SVC) and deep vascular complex (DVC).

### 3.5 Multivariable Modeling and Feature Selection

Of the clinical information collected from our AD patients, ten features were selected based on non‐zero coefficients from LASSO logistic regression analysis (Figure 2). The selected features included plasma Aβ42, plasma Aβ42/40, plasma p‐tau217, PSMD, amyloid PET SUVR, hippocampal volume, MTA, retinal FD, SVC, and DVC. In the sensitivity analysis additionally adjusted for hyperlipidemia, disease duration, and MoCA, PSMD (OR, 7.693; 95% CI, 1.437–41.178; *p* = 0.017), SVC (OR, 0.123; 95% CI, 0.022–0.687; *p* = 0.017), SUVR (OR, 5.228; 95% CI, 1.993–13.712; *p* < 0.001), and hippocampal volume (OR, 0.110; 95% CI, 0.025–0.492; *p* = 0.004) remained independently associated with APOE ε4 status, whereas the other selected features were no longer statistically significant (Table S2).

**FIGURE 2:**
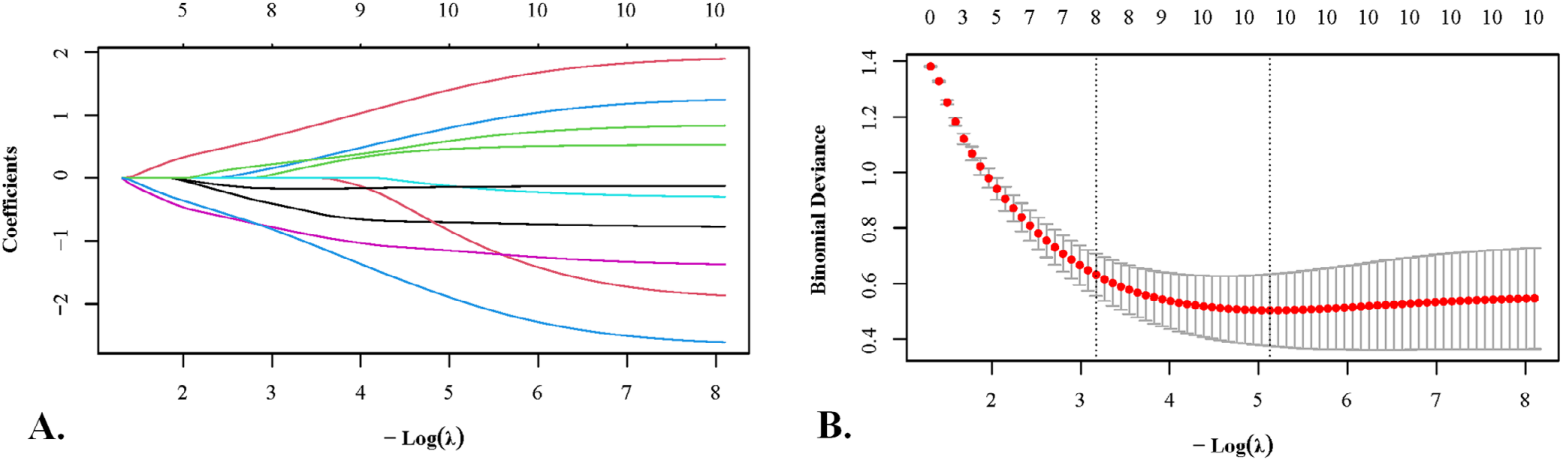
Feature Selection via LASSO Logistic Regression. The process of narrowing down variables using a Least Absolute Shrinkage and Selection Operator (LASSO) binary logistic regression is illustrated through the following two components: (A) Coefficient Distribution and Lambda Evolution. A coefficient profile was generated to track how the ten features fluctuate against the log lambda sequence. This visualization demonstrates how the LASSO algorithm applies a penalty to the model, gradually shrinking less impactful coefficients toward zero as the tuning parameter increases. (B) Optimal Parameter Selection via Cross‐Validation To identify the most effective tuning parameters, a five‐fold cross‐validation method was employed, focusing on the minimum criterion. Validation Metrics: The relationship between the partial likelihood deviance (binomial deviance) and the logarithmic lambda values was plotted. Optimal Thresholds: Two vertical dashed lines were used to mark critical points. Utilizing the optimal lambda value, the model successfully refined the feature set to four non‐zero coefficients, effectively eliminating redundant noise from the final model.

### 3.6 Development of Nomogram

Based on the data obtained, a multivariate logistic regression analysis showed that PSMD (OR, 4.255, 95% CI 1.165 to 15.543, *p* = 0.028), SVC (OR, 0.121, 95% CI 0.019 to 0.766, *p* = 0.024), SUVR (OR, 5.206, 95% CI 2.098 to 12.918, *p* < 0.001), and hippocampal volume (OR, 0.153, 95% CI 0.041 to 0.571, *p* = 0.005) were independent contributors to genotype‐associated biological variation. No significant multicollinearity was detected among retained predictors (Figure 3). After additionally adjusting for disease duration and MoCA scores, these selected features remained as shown in Table S2. These variables were incorporated into a nomogram model for AD classification based on APOE ε4 genotype.

**FIGURE 3:**
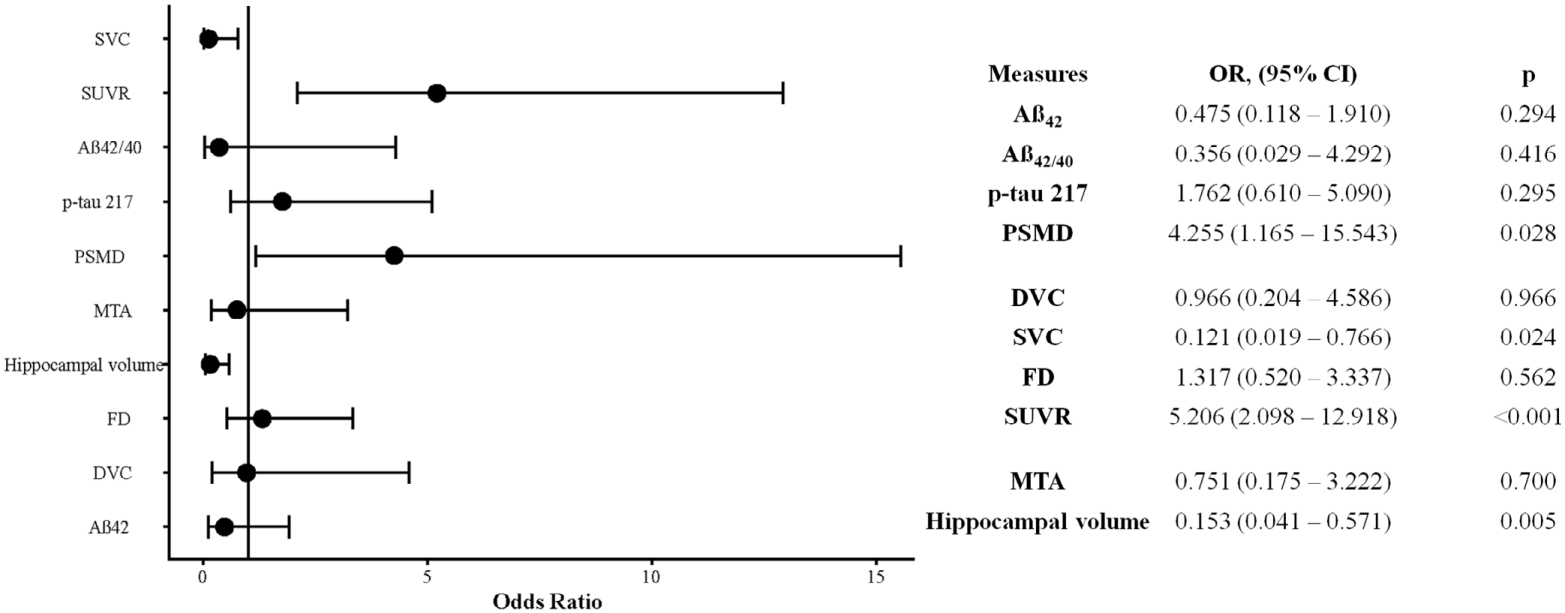
Multivariate logistic regression analyses and forest plots for assessing measures associated with APOE ε4 on features selected by the LASSO regression model. Based on the data obtained using LASSO analysis. PET SUVR, PSMD, hippocampal volume, and SVC were independently associated with APOE ε4 genotype.

A nomogram based solely on neuroimaging measures (conventional model) demonstrated a C‐index of 0.904; a bootstrap‐corrected C‐index of 0.861. We further added SVC, and the nomogram demonstrated a C‐index of 0.954 and a bootstrap‐corrected C‐index of 0.932, indicating the additive value of retinal biomarkers. The calibration curves for the probability of AD based on APOE ε4 genotype using this nomogram indicated good agreement between the additive value of retinal biomarkers and observation in our cohort, which was also confirmed by the Hosmer‐Lemeshow test (*p* = 0.250). DCA revealed that the nomogram provided a higher net clinical benefit than the conventional imaging model across a wide range of threshold probabilities, indicating superior clinical utility of the proposed model (Figure 4).

**FIGURE 4:**
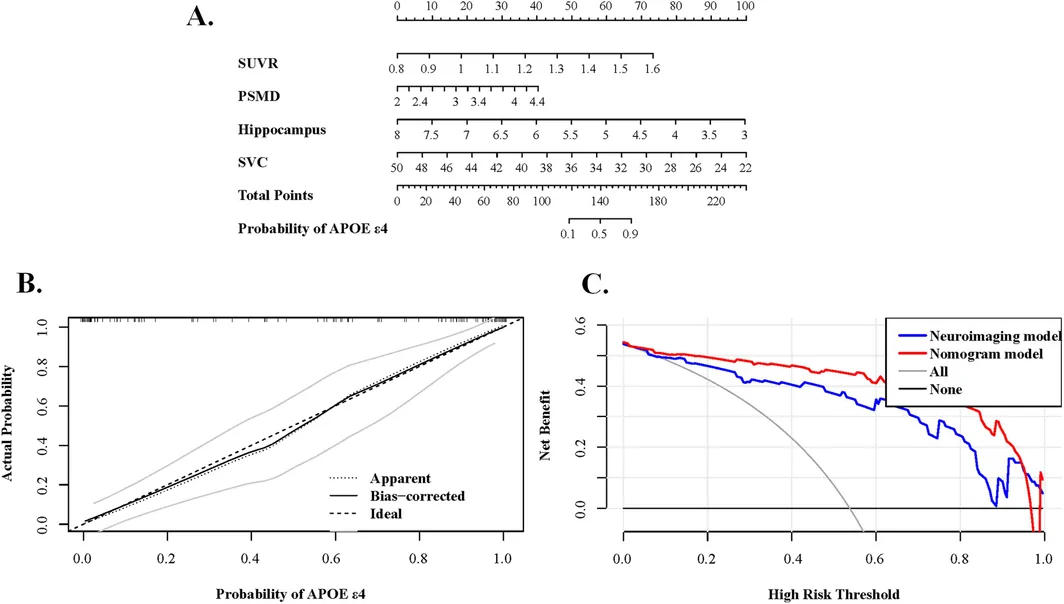
Developed a nomogram and performance measures for the APOE ε4 genotype in AD. This figure illustrates the development and validation of a diagnostic nomogram integrating neuroimaging and retinal biomarkers to provide additive value for genotype‐specific biological stratification in biomarker‐confirmed AD. (A) Nomogram: A clinical tool for genotype‐specific biological stratification in biomarker‐confirmed AD. Points are assigned based on values for Amyloid PET Standardized Uptake Value Ratio (SUVR), peak width of skeletonized mean diffusivity (PSMD), hippocampal volume, and retinal microvascular complexity (SVC). (B) Calibration Curve: The calibration plot assesses the agreement between predicted and actual probabilities of the APOE ε4 genotype in AD. (C) Decision Curve Analysis (DCA): DCA demonstrates the clinical net benefit of using the combined nomogram model (red line) compared to a model using neuroimaging alone (blue line). The nomogram model provides superior net benefit across a wide range of high‐risk threshold probabilities.

### 3.7 Nomogram‐Based Probability of Differentiating AD With APOE ε4 Carriers From Non‐Carriers

Model performance was further validated using receiver operating characteristic (ROC) analysis, as shown in Figure 4. Compared to the conventional neuroimaging model (AUC = 0.902, Table 2), the nomogram achieved an AUC of 0.951, indicating the novel nomogram had a higher ability to define a multimodal vascular–neurodegenerative phenotype associated with APOE ε4 and demonstrating the additive value of retinal biomarkers. Table S3 shows the model performance after additionally adjusting for disease duration and MoCA scores.

**TABLE 2:** ROC analysis of the nomogram.

Measures	AUC	Youden's index	Sensitivity	Specificity
Neuroimaging	0.902	0.703	80.3	90.0
SVC	0.855	0.583	80.3	78.0
Neuroimaging + SVC	0.951	0.812	85.2	96.0

Abbreviations: AUC, area under the curve; SVC, superficial vascular complex.

## 4 Discussion

In this multimodal study of biomarker‐confirmed AD, we investigated whether retinal microvascular imaging provides complementary information for characterizing APOE ε4‐associated biological heterogeneity beyond established plasma and neuroimaging biomarkers. APOE ε4 carriers demonstrated greater cerebral amyloid burden, more severe white matter microstructural injury, smaller hippocampal volume, and reduced retinal microvascular density compared with non‐carriers. Importantly, retinal SVC density remained independently associated with APOE ε4 status after accounting for established neuroimaging biomarkers and further improved the performance of a multimodal phenotyping model. Collectively, these findings suggest that retinal microvascular alterations are associated with the neurovascular phenotype linked to APOE ε4 in biologically defined AD and provide complementary information beyond conventional markers of amyloid deposition and neurodegeneration.

APOE ε4 is well established as the strongest common genetic determinant of sporadic AD and is associated with accelerated amyloid accumulation, greater tau burden, earlier neurodegeneration, and increased susceptibility to cerebrovascular dysfunction [7]. Consistent with previous studies, APOE ε4 carriers in our cohort exhibited higher amyloid PET SUVR, greater PSMD, and smaller hippocampal volumes, supporting the concept that APOE ε4 influences multiple biological pathways rather than amyloid pathology alone [5, 42, 43]. Our findings extend this evidence by demonstrating that retinal microvascular abnormalities accompany these cerebral alterations, suggesting that vascular changes occur concurrently across both central and retinal microvascular beds in biomarker‐confirmed ad [5, 21, 22].

The retina provides a unique opportunity to evaluate neurovascular integrity because it shares embryological origin, anatomical organization, and microvascular characteristics with the cerebral circulation. Increasing evidence indicates that retinal vascular density and vascular complexity are associated with SVD, cognitive impairment, and neurodegeneration [17, 20, 21, 22]. In the present study, APOE ε4 carriers demonstrated significantly lower SVC and DVC vessel densities together with reduced retinal vascular fractal dimension. These observations support an association between APOE ε4 and widespread microvascular alterations involving both the brain and retina. Nevertheless, because of the cross‐sectional design, our findings should be interpreted as demonstrating biological associations rather than causal mechanisms. Whether retinal vascular alterations represent downstream consequences of cerebral pathology, parallel manifestations of shared vascular dysfunction, or both requires confirmation in longitudinal and mechanistic studies.

An important objective of this study was to determine whether retinal vascular biomarkers provide incremental biological information beyond conventional plasma and neuroimaging measures. We therefore evaluated the additive contribution of retinal SVC density within a multimodal framework that incorporated amyloid burden, hippocampal volume, and PSMD. Although the neuroimaging model already demonstrated excellent discrimination, the addition of retinal SVC modestly improved model performance and calibration. These findings suggest that retinal imaging captures a component of APOE ε4‐associated biological heterogeneity that is not completely reflected by established imaging biomarkers. Rather than serving as a surrogate for APOE genotyping, retinal vascular imaging should be viewed as a complementary biomarker that may improve characterization of vascular involvement within biologically confirmed AD.

The present findings should also be interpreted in light of differences in baseline disease severity between APOE ε4 carriers and non‐carriers. APOE ε4 carriers exhibited lower baseline cognitive performance, raising the possibility that retinal and neuroimaging abnormalities could partially reflect more advanced disease rather than genotype‐associated biological differences alone. To address this concern, we performed additional sensitivity analyses incorporating cognitive performance as an additional covariate. The association between retinal microvascular changes and APOE ε4 status remained significant after adjustment, supporting the robustness of the observed retinal findings. Nevertheless, we cannot completely exclude residual confounding by disease severity, and future studies involving cognitively matched cohorts or longitudinal follow‐up will be necessary to further disentangle genotype‐specific effects from disease progression.

Similarly, because retinal microvasculature is influenced by systemic vasculature, we additionally evaluated the potential confounding effect of cardiovascular risk factors. The association between APOE ε4 and retinal microvascular changes remained significant following additional adjustment for hyperlipidemia, suggesting that the retinal vascular alterations observed in this study are unlikely to be explained solely by differences in conventional vascular risk factors. However, residual confounding from unmeasured vascular variables cannot be completely excluded.

The biological significance of reduced retinal vascular density in APOE ε4 carriers remains speculative. Experimental studies have shown that APOE ε4 contributes to endothelial dysfunction, impaired vascular repair, blood–brain barrier disruption, and reduced cerebrovascular resilience [42]. These mechanisms could plausibly contribute to the concurrent cerebral and retinal vascular alterations observed in the present study. However, our cross‐sectional findings cannot establish mechanistic pathways, and alternative explanations, including secondary vascular changes resulting from more advanced neurodegeneration, should also be considered. Future longitudinal investigations incorporating blood–brain barrier imaging, cerebrovascular reactivity, glymphatic biomarkers, and serial retinal imaging may further clarify these relationships.

Several strengths enhance the validity of this study. Participants fulfilled contemporary biological diagnostic criteria for AD using amyloid PET confirmation, underwent standardized multimodal phenotyping including plasma biomarkers, MRI, diffusion imaging, OCTA, and quantitative CFP, and retinal image quality was assessed according to internationally recognized OCTA reporting recommendations. Furthermore, retinal microvascular analyses accounted for inter‐eye correlation using GEE, while candidate predictors were initially reduced using penalized regression before construction of an interpretable multivariable model.

From a clinical perspective, these findings suggest that retinal vascular imaging may have value as a complementary component of multimodal phenotyping rather than as an independent diagnostic substitute for APOE genotyping or established AD biomarkers. In our cohort, retinal SVC density contributed additional information to a neuroimaging‐based model for distinguishing APOE ε4 carriers from non‐carriers, and this incremental model performance remained evident after additional adjustment for clinical factors and cognitive performance. This may be relevant because retinal imaging is non‐invasive and readily repeatable, potentially providing an accessible measure of microvascular involvement that could complement cerebral imaging and blood‐based biomarkers. However, the present study does not establish that retinal vascular alterations predict cognitive decline or longitudinal disease progression. The additional adjustment for MoCA and disease duration supports the robustness of the observed cross‐sectional associations but does not establish temporal independence from disease severity. Prospective longitudinal studies with repeated retinal imaging, cognitive assessments, and plasma or neuroimaging biomarkers will therefore be necessary to determine whether retinal vascular measures are associated with subsequent cognitive trajectories, disease progression, or treatment response.

Several limitations should also be acknowledged. First, the study was conducted at a single center with a relatively modest sample size, increasing the possibility of model optimism despite penalized feature selection and bootstrap internal validation. Independent external validation in larger multicenter cohorts will be essential before broader application of these findings. Second, although the final multivariable model included only variables retained following LASSO feature selection, the overall sample size limited model complexity and statistical power. Third, the cross‐sectional design precludes inference regarding temporal relationships or causality. Fourth, although major vascular comorbidities and cognitive performance were considered in sensitivity analyses, residual confounding cannot be completely excluded. Finally, the present findings are specific to biomarker‐confirmed AD and should not be generalized to other neurodegenerative disorders without further investigation.

In conclusion, retinal microvascular alterations are associated with APOE ε4‐related biological heterogeneity in biomarker‐confirmed AD. Retinal SVC density provided complementary information beyond established plasma and neuroimaging biomarkers and modestly improved multimodal characterization of genotype‐associated neurovascular phenotypes. These findings support the potential role of retinal vascular imaging as a non‐invasive complementary biomarker for investigating vascular contributions to AD biology and provide a foundation for future longitudinal studies evaluating retinal imaging as a marker of disease progression, therapeutic response, and precision phenotyping.

## Author Contributions

Study concept and design: W.R.K., H.C. Data acquisition: H.C., C.W., H.W., H.X. Data analysis and interpretation: W.R.K., H.C., L.C., Y.W. Drafting of the manuscript: W.R.K., H.C., Z.W., L.C., Y.W. Critical review of the manuscript: W.R.K., H.C., Y.W., L.C., C.W. All authors reviewed and approved this version of the manuscript.

## Funding

The authors have nothing to report.

## Ethics Statement

The protocols of the study were approved by the Ethics and Review Committee of the First Affiliated Hospital of Wenzhou Medical University (Ethics Number: KY2021‐153).

## Consent

All participants gave their written informed consent. Ethical approval was granted by the Ethics Committee of The First Affiliated Hospital of Wenzhou Medical University (Ethics Number: KY2021‐153).

## Conflicts of Interest

The authors declare no conflicts of interest.

## Supporting information


**Figure S1:** Multimodal biomarkers used for APOE ε4 stratification in Alzheimer's disease.
**Figure S2:** Flow chart of patient selection.
**Table S1:** Comparison of demographics and clinical information of AD based on APOE ε4 genotype.
**Table S2:** Multivariate logistic regression analyses for assessing measures associated with APOE ε4 on features selected by the LASSO regression model. The data shown here were additionally adjusted for hyperlipidemia, duration and cognitive performance.
**Table S3:** ROC analysis of the nomogram after additionally adjusting for hyperlipidemia and cognitive performance.

## Data Availability

The dataset generated and analyzed for this study will be made available by the corresponding author upon reasonable request.
