# Supplementary material for: Multimodal Characterization of APOE ε4‐Associated Neurovascular Heterogeneity Using Retinal, Plasma, and Neuroimaging Biomarkers in Alzheimer's Disease
Source: CNS Neurosci Ther. 2026 Oct 24;32(10):e71220. doi: 10.1002/cns.71220 (PMC13695082; doi:10.1002/cns.71220)
Supplement: Supplementary file 1 [file CNS-32-e71220-s001.docx]

**Supplementary Figure 1. Multimodal biomarkers used for APOE ε4 stratification in Alzheimer’s disease.**

(A) PET/MR imaging. Panel I shows representative amyloid positron emission tomography (PET) images demonstrating elevated cerebral amyloid deposition, expressed as standardized uptake value ratio (SUVR). Panel II illustrates structural magnetic resonance imaging (MRI) with medial temporal lobe atrophy (MTA) scoring, highlighting hippocampal atrophy and enlargement of surrounding cerebrospinal fluid spaces. Panel III presents diffusion tensor imaging (DTI)–derived mean diffusivity maps. Panel IV depicts the skeletonized white matter framework and corresponding histogram used to derive peak width of skeletonized mean diffusivity (PSMD).

(B) Blood-based biomarkers. Schematic representation of plasma biomarkers measured in this study, including amyloid-β (Aβ) species, tau isoforms, neurofilament light chain (NfL), and glial fibrillary acidic protein (GFAP).

(C) Retinal vascular imaging. Panel I shows a macula-centered color fundus photograph. Panel II illustrates retinal vascular skeletonization and grid-based box-counting analysis used to quantify retinal vascular fractal dimension. Panel III displays optical coherence tomography angiography (OCTA) images of the superficial vascular complex (SVC) and deep vascular complex (DVC), along with corresponding structural OCT B-scan indicating segmentation boundaries.

**Supplementary Figure 2. Flow chart of patient selection.**

**Supplementary Table 1. Comparison of demographics and clinical information of AD based on APOE ε4 genotype**

|  | **Non-carrier**  **N = 42** | **Carrier**  **N = 42** | **p** |
| --- | --- | --- | --- |
| Age, years | 66.57 ± 6.85 | 68.12 ± 6.24 | 0.282 |
| Males, n (%) | 13 (30.95%) | 14 (33.33%) | 1 |
| Duration, years | 3 [2, 4] | 3 [2, 4] | 0.984 |
| Hyperlipidemia, n (%) | 21 (50.0%) | 13 (30.95%) | 0.120 |
| Diabetes mellitus, n (%) | 11 (26.19%) | 12 (28.57%) | 1 |
| Hypertension, n (%) | 15 (35.71%) | 18 (42.86%) | 0.655 |
| Education, years | 6 [6, 9] | 9 [6, 11] | 0.875 |
|  |  |  |  |
| Global cognitive measures |  |  |  |
| MMSE | 20 [17, 23] | 15 [12, 20] | 0.005 |
| MoCA | 14 [11, 16] | 11 [8, 13] | 0.002 |
|  |  |  |  |
| *Blood plasma biomarkers* |  |  |  |
| Aß_42_, pg/mL | 7.28 ± 1.66 | 5.84 ± 1.67 | <0.001 |
| Aß_42/40_ | 0.06 ± 0.02 | 0.05 ± 0.01 | 0.009 |
| p-tau 181, pg/mL | 8.67 ± 3.90 | 10.24 ± 4.17 | 0.112 |
| p-tau 217, pg/mL | 9.23 ± 3.48 | 11.10 ± 4.17 | 0.047 |
| p-tau 217/ Aß_42_ | 1.39 ± 0.76 | 2.17 ± 1.29 | 0.004 |
| NfL, pg/mL | 64.66 ± 54.54 | 74.47 ± 45.69 | 0.426 |
| GFAP, pg/mL | 225.62 ± 105.91 | 288.73 ± 133.76 | 0.037 |
|  |  |  |  |
| **Ophthalmic imaging metrics* |  |  |  |
| Fundus vessels, FD | 1.35 ± 0.08 | 1.26 ± 0.11 | <0.001 |
| SVC, % | 40.84 ± 3.21 | 33.83 ± 4.24 | <0.001 |
| DVC, % | 51.06 ± 3.55 | 45.06 ± 8.30 | <0.001 |
|  |  |  |  |
| *Quantitative PET measure* |  |  |  |
| SUVR | 1.15 ± 0.18 | 1.31 ± 0.11 | <0.001 |
|  |  |  |  |
| *MRI measures* |  |  |  |
| MTA | 1 [1, 2] | 2 [1, 2] | <0.001 |
| Hippocampal volume, mm^3^ | 6.21 ± 0.72 | 5.36 ± 0.71 | <0.001 |
| PSMD, ×10^-4^ mm/s | 2.69 ± 0.37 | 2.99 ± 0.52 | <0.001 |

MMSE, Mini-Mental State Examination; MoCA, Montreal Cognitive Assessment; NfL, neurofilament light chain; PSMD, peak width of skeletonized mean diffusivity; SVC, superficial vascular complex; DVC, deep vascular complex; SUVR, standardized uptake value ratio.

*Generalized estimating equation models were used to compare the two groups while adjusting for age, sex, hypertension, diabetes mellitus, education and intereye dependencies

**Supplementary Table 2. Multivariate logistic regression analyses for assessing measures associated with APOE ε4 on features selected by the LASSO regression model.** The data shown here were additionally adjusted for hyperlipidemia, duration and cognitive performance.

| **Measures** | **OR (95% CI)** | **p** |
| --- | --- | --- |
| Aß_42_ | 0.461 (0.110 – 1.925) | 0.288 |
| Aß_42/40_ | 0.591 (0.049 – 7.197) | 0.680 |
| p-tau 217 | 2.674 (0.760 – 9.414) | 0.125 |
| PSMD | 7.693 (1.437 – 41.178) | 0.017 |
| DVC | 0.971 (0.200 – 4.720) | 0.971 |
| SVC | 0.123 (0.022 – 0.687) | 0.017 |
| FD | 1.317 (0.516 – 3.364) | 0.565 |
| SUVR | 5.228 (1.993 – 13.712) | <0.001 |
| MTA | 0.786 (0.190 – 3.254) | 0.740 |
| Hippocampal volume | 0.110 (0.025 – 0.492) | 0.004 |

PSMD, peak width of skeletonized mean diffusivity; SVC, superficial vascular complex; DVC, deep vascular complex; SUVR, standardized uptake value ratio.

**Supplementary Table 3. ROC analysis of the nomogram after additionally adjusting for hyperlipidemia and cognitive performance**

| **Measures** | **AUC** | **Youden’s index** | **Sensitivity** | **Specificity** |
| --- | --- | --- | --- | --- |
| Neuroimaging | 0.906 | 0.723 | 80.3 | 92.0 |
| SVC | 0.880 | 0.702 | 90.2 | 80.0 |
| Neuroimaging + SVC | 0.955 | 0.845 | 88.5 | 96.0 |

AUC: area under the curve; SVC, superficial vascular complex.
